# A Twitter-facilitated professional learning community: online participation, connectedness, and satisfaction

**DOI:** 10.1186/s12909-022-03639-6

**Published:** 2022-07-27

**Authors:** Binbin Zheng, Gary Beck Dallaghan

**Affiliations:** 1 Faculty of Medicine, The University of Hong Kong, Room 515, 5/F, William MW Mong Block, 21 Sassoon Road, Pokfulam, Hong Kong; 2 School of Medicine, University of North Carolina, Chapel Hill, USA

**Keywords:** Twitter, Professional learning community, Cognitive presence, Social presence, Moderator presence

## Abstract

**Background:**

Twitter has gained increasing popularity and attention as a professional learning environment to share knowledge, exchange information, make connections, and build networks. To evaluate the effectiveness of Twitter-facilitated online discussions, a community of inquiry framework could be used with the three key elements of online environments: cognitive presence, social presence, and teaching presence. This study aims to explore how medical educators participate in synchronous online discussions on Twitter using #MedEdChat, and how participants’ perceptions toward the three presences, sense of connectedness and interactions influenced their online satisfaction.

**Methods:**

A survey invitation was emailed using the medical education email list DR-ED and was posted during the weekly Twitter conversations in December 2020, to solicit participants who have been involved in any kind of #MedEdChat activities (i.e., read transcripts or directly participate in discussions).

**Results:**

A total of 68 people responded. Through descriptive analysis and path analysis, we found that almost half of the survey respondents were lurkers on #MedEdChat who read others’ tweets or transcripts. In addition, participants mainly used Twitter for resource sharing, collaborating with others, and networking. Participants rated teaching (i.e., moderator) presence the highest, followed by overall satisfaction, cognitive presence, sense of connectedness, social presence, and interactions. Among them, sense of connectedness and cognitive presence were significantly associated with participants’ overall satisfaction.

**Conclusions:**

This study provided significant implications for using Twitter as a professional learning community to conduct online discussion activities. Facilitators could think of ways to improve participation by providing tutorials on how to participate on Twitter discussions, introduce or ask new participants to introduce themselves, facilitate discussion with intriguing questions, and invite medical educators of different roles as well as medical students and residents to join to bring in diverse perspectives.

## Background

The COVID-19 pandemic has accelerated the growth in importance of social media in our daily and professional lives [10, 17, 18]. One of the most popular social-media platforms used by professionals is Twitter. Compared to other types of social media, Twitter allows its users to post short messages (no longer than 280 characters) and use hashtags (#) to highlight a topic or a community [6]. Because of the unique characteristic of allowing users to send short messages, Twitter is also considered a type of microblogs that allows information dissemination at users’ fingertips, and is the most prominent microblogging platform on the market [11]. Twitter has been increasingly used by educators to establish communities of practice (CoPs [32]); via social interaction [23], the members of which connect with like-minded professionals from all over the world, conversing with them and sharing information [11, 33]. With the affordances of facilitating communication both synchronously and asynchronously, Twitter has been proven to be beneficial for community building and collaborative learning [7], and to enable democratization by allowing anyone to contribute [30]. As a result, a CoP might be utilized to support professional growth among online participants who share a common knowledge area, increasing networking possibilities while minimizing the geographic barriers to interprofessional engagement that many professionals experience [12, 35]. Another benefit of Twitter-enabled CoPs is that they allow educators to engage with others outside of their typical work settings, giving them a sense of professional belonging during earlier stages of their careers than might otherwise be possible [5].

The use of Twitter for professional development among health professionals has received considerable attention lately because of the ease with which its users can hold both synchronous and asynchronous discussions, by utilizing hashtags (#) to signal content. A systematic review by Sterling et al. [28] noted that Twitter was the most frequently used platform to promote conference themes and presentation content at medical conferences using hashtags. Theme-based discussions are also held on Twitter for a wide variety of other educational purposes. For example, to develop clinical environments for work-based learning (WBL), two UK universities hosted a series of Twitter chats among academics, healthcare professionals, and students, which collectively formed a CoP focused on WBL [2]. On the other hand, while healthcare professionals generally hold positive views about the use of Twitter and other social media to distribute knowledge, there is skepticism about the reliability of the information conveyed via such platforms, and thus, a culture of trust and collectivism must be established and encouraged if social media are to realize their potential for knowledge exchange and community-building [23].

To better understand and facilitate learning in online community, the Community of Inquiry (CoI) framework [8] is widely used to evaluate a given online learning environment using three elements: cognitive presence, social presence, and teaching presence. Cognitive presence refers to “the extent to which learners are able to construct meaning through sustained reflection and discourse”; social presence, to “the ability of participants to project their personal characteristics into the community”; and teaching presence, to “the design, facilitation and direct instruction of the educational experience.” Research has suggested that these three core elements of online environments are important for learner satisfaction, sense of belonging, and learning outcomes (e.g., [1, 22, 34]). The framework has also been used in Twitter studies to examine these three types of online presences. For example, Solmaz [27] found that pre-service teachers reported positive perceptions towards use of Twitter to establish and maintain a community of inquiry, indicating high social and teaching presences on Twitter discussions. Similarly in another study [21], students demonstrated high social presences on Twitter, while another domain of presence – learning presence, proposed by Shea and Bidjerano [25] as an additional constuct to CoI, defined as students’ ability to self- and co-regulate learning behaviors – was also found to be strong on Twitter.

However, research focused on applying CoI with Twitter use as a professional learning community for medical educators is still scant. As a result, this study explores how medical educators participated and engaged in discussions in a Twitter-facilitated professional learning activity using #MedEdChat, and what CoI elements influenced participant satisfaction. Three questions were explored in this study:

1. What was the participation pattern on #MedEdChat?

2. How did participants’ perceptions towards the online community, sense of connectedness, and online interaction influence their satisfaction on #MedEdChat?

3. How did #MedEdChat influence participants as medical educators?

## Methods

### Context

This study was approved by the Institutional Review Board in the designated university. As a medical-education-focused hashtag, #MedEdChat runs weekly conversations around certain medical education topics every Thursday night at 9 pm EST, moderated by the Alliance for Clinical Education. The #MedEdChat began in May, 2011 and currently has over 9000 followers throughout the world. Because the topics on #MedEdChat are generally not controversial, conversations on it tend to contain only polite discourse, and there is no backchannel screening of posts. However, the moderator does post instructions at the beginning of each week’s discussion, reminding people to use the #MedEdChat hashtag and that “all of your tweets during #MedEdChat are your own”. During the one-hour live chat, the moderator copies all the links people share and pastes them into a Word document. After each live chat, the moderator uses the website symplur.com to generate a transcript of all #MedEdChat tweets posted from 9 pm EST on that day up until the moment the transcript is generated. Next, the links are inserted into the transcript, which is then posted publicly on the Alliance for Clinical Education website in the form of a single PDF file.

### Data collection

A survey invitation was emailed using the medical education email list, DR-ED (3591 subscribers at the time of the study), and was posted during the weekly Twitter conversations in December 2020, to solicit participants who have been involved in any kind of #MedEdChat activities (i.e., read transcripts or directly participate in discussions). The survey was designed to better understand participants’ experience using #MedEdChat, i.e., their frequency and degree of participation and discussion, perceptions toward the online community, sense of connectedness, online peer interaction, and online satisfaction. Specifically, two demographic-information questions, and seven questions regarding their Twitter usage and #MedEdChat participation, were developed by the researchers themselves (see Appendix). Eleven survey questions about perceptions towards moderator presence, social presence, and cognitive presence were adopted from the Community of Inquiry framework [1, 9]. Three survey questions about the sense of connectedness were adopted from the Classroom Community Scale [24]. In addition, three survey questions related to online peer interactions and three questions related to online satisfaction were adopted from Kuo et al. [16]. The questions about the three CoI presences, sense of connectedness, interaction, and satisfaction were all responded to on the same five-point Likert scale, ranging from 1 = “strongly disagree” to 5 = “strongly agree”. The survey also included two open-ended questions: one asked about what individuals gained from participating on #MedEdChat, and the other asked about how has participation on #MedEdChat influenced their work as a medical educator. All participation in the survey was anonymous and voluntary. Given the nature of listserv and Twitter, an accurate response rate could not be calculated. As a result, a total of 68 respondents were included in this study.

### Data analysis

Descriptive statistics were used to answer the first research question about participants’ overall participation on #MedEdChat, including frequencies of visits, levels of participation, interested topics, and professional purposes of using #MedEdChat. To answer the second question about the relationship among online community perceptions and overall satisfaction with the use of #MedEdChat, path analysis [29] was adopted with online satisfaction as the dependent variable. Predictors included three types of online presences (i.e., moderator presence, social presence, and cognitive presence), sense of connectedness, and perceived peer interaction. Social presence and cognitive presence were entered during the first step of path analysis. Moderator presence was added in the second step, while sense of connectedness was added in the third step, and interaction was added in the last step. Finally, to answer the third research question about how using #MedEdChat influenced participants as medical educators, conventional content analysis [13] with a bottom-up coding approach was used to extract themes from the data.

## Results

### Participation pattern

Among the 68 participating respondents, 70.6% were female (*N* = 48). Almost half of them (*N* = 33, 48.5%) are clinician educators, followed by 9 social science educators and 6 basic science educators. Twenty participants identified themselves as other roles in medical education, including medical education administrators/staff, faculty developers/educators, and librarians. None of them were medical students, residents, or fellows. Most of the participants (67.8%, *N* = 42) have more than 3 years’ experience using Twitter, and the frequency varies. Survey participants (29.5%) mentioned using Twitter only a few times a year, 26.2% using it weekly, 23% started using it daily, while 16.4% mentioned they would use Twitter multiple times a day (see Table 1 for the result of the descriptive statistics).

**Table 1:** Descriptive statistics of participants’ general information

	Number	Percentage
**Role in Medical Education**		
Clinician educator	33	48.5%
Basic science educator	6	8.8%
Social science educator	9	13.2%
Medical student	0	0%
Resident/Fellow	0	0%
Other	20	29.4%
Total	68	100%
**Gender**		
Female	48	71%
Male	20	29%
Non-binary	0	0%
Total	68	100%
**Twitter usage history**		
Less than six months	8	12.9%
Less than one year	1	1.6%
Less than 2 years	6	9.7%
Less than 3 years	5	8%
Three years or more	42	67.7%
Total	62	100%
**Twitter usage frequency**		
Multiple times a day	10	16.4%
Daily	14	23%
Weekly	16	26.2%
Monthly	3	4.9%
Only a few times a year	18	29.5%

In terms of participants’ involvement in #MedEdChat, the largest percentage (46.8%, *N* = 29) mentioned that they never participated directly on #MedEdChat, but would read the transcripts. Of those that directly participate, 22.6% mentioned participating once every 2-3 months, 17.7% mentioned that they participated once or twice a year. Only four mentioned that they participated monthly and another four mentioned their participation frequency is 2-3 times a month. None of the respondents participated weekly on #MedEdChat.

Participants indicated the type of participation with the #MedEdChat. Based on their responses, their participation type from top to low are: lurkers (who read others’ tweets on #MedEdChat) > like others’ tweets on #MedEdChat > retweet others’ tweets on #MedEdChat > respond to others’ tweets > post original tweets on #MedEdChat. Among the six topics listed, participants mentioned that they were most likely to see topics on faculty development, followed by medical student learning, medical education scholarship, curriculum, assessment, and technology integration. When asking about the professional purpose of using #MedEdChat, participants rated “resource sharing” as the highest, followed by “collaborating with other educators”, “networking”, “providing own opinions/insights”, and “emotional support”.

### Satisfaction and influencing factors on #MedEdChat

We asked about participants’ perceptions of the online community (i.e., moderator presence, social presence, and cognitive presence), sense of connectedness, interaction, and their overall satisfaction with #MedEdChat. Descriptive statistics were presented in Table 2. Overall, participants had the most positive attitudes towards moderator presence (M = 4.07, SD = .83), especially on the survey item “The moderator provided clear instructions on how to participate in the #MedEdChat discussions” (M = 4.27, SD = .94). Following that, participants had about the same agreement towards social presence (M = 3.61, SD = .91), cognitive presence (M = 3.84, SD = .62), and sense of connectedness (M = 3.81, SD = .84). However, participants had less positive attitudes towards the online interactions happened on #MedEdChat (M = 3.01, SD = .86), while the overall satisfaction was rated quite high (M = 4.02, SD = .88). Among variables on satisfaction, participants agreed the most with “I would recommend #MedEdChat to other medical educators” (M = 4.27, SD = .95). In addition, all these variables are highly correlated with one another (see Table 3).

**Table 2:** Descriptive statistics of the key variables about participants’ perceptions

Variable	Observation	Mean	S.D.	Min	Max
Moderator presence	49	4.07	.83	1	5
Social presence	49	3.61	.91	1.25	5
Cognitive presence	50	3.84	.62	1.5	5
Sense of connectedness	48	3.81	.84	1.3	5
Interaction	47	3.01	.86	1	5
Overall satisfaction	51	4.02	.88	1.3	5

**Table 3:** Correlations among key variables

Variables	Moderator presence	Social presence	Cognitive presence	Sense of connectedness	Interaction	Overall satisfaction
Moderator presence	1.000					
Social presence	0.607***	1.000				
Cognitive presence	0.568***	0.707***	1.000			
Sense of connectedness	0.662***	0.768***	0.740***	1.000		
Interaction	0.384***	0.686***	0.543***	0.522***	1.000	
Overall satisfaction	0.689***	0.717***	0.774***	0.831***	0.523***	1.000

*** p < 0.01, ** p < 0.05, * p < 0.1

Multiple regression analysis was used to examine how participants’ perception of the online community, sense of connectedness, and interaction influenced their satisfaction (see Table 4). Model 1 included cognitive presence, Model 2 added social presence, Model 3 added moderator presence, Model 4 further included sense of connectedness, and Model 5 added interaction. As we can see from the final model that, when included all variables in, only cognitive presence (coeff. = .39, *p* < .05) and sense of connectedness (coeff. = .51, *p* < .001) had significant effect on overall satisfaction. Social presence, moderator presence, and interaction had no significant effects on overall satisfaction.

**Table 4:** Multiple regression with satisfaction as the predictor variable

	Outcome: Satisfaction				
	Model 1	Model 2	Model 3	Model 4	Model 5
Cognitive presence	1.059*** (8.38)	0.737*** (4.38)	0.613*** (3.62)	0.404* (2.49)	0.389* (2.42)
Social presence		0.315**	0.220	0.0401	0.0259
		(2.76)	(1.88)	(0.35)	(0.20)
Moderator presence			0.271*	0.174	0.129
			(2.32)	(1.62)	(1.18)
Sense of connectedness				0.476***	0.511***
				(3.53)	(3.84)
Interaction					0.0332
					(0.33)
Constant	−0.0172	0.0751	−0.198	−0.162	−0.124
	(−0.03)	(0.16)	(−0.43)	(− 0.39)	(− 0.30)
N	49	48	48	48	47
R-square	0.60	0.66	0.70	0.76	0.78

t statistics in parentheses

* p < 0.05 ** p < 0.01 *** p < 0.001

### Gains and influences for medical educators

From the open-ended questions, we received 35 comments about gains from participating on #MedEdChat. Fifteen comments mentioned the biggest gain is resources. Because resources that were discussed on #MedEdChat were usually included in the final transcript, participants felt that those resources were helpful and valuable for their own work and scholarship. Moreover, 13 comments were about new ideas and opinions that they were able to hear about and learn from. Five mentioned they gained the most that #MedEdChat created a sense of community “with like-minded educators/researchers”, and helped “get to reach outside our silos (whether that’s institutional, clinical specialty, or geography)”. Finally, 2 mentioned connections they can make is the biggest gain, especially with “people I admire but not personally connected with”.

Among the 33 comments responding to the question “how has #MedEdChat influenced your work as a medical educator”, many mentioned that they can apply the “new perspectives” into teaching or pass on to colleagues. Even more, one participant voiced that “sharing the links and transcripts adds to my credibility when offering ideas within my institution”. Another common theme is that participating on #MedEdChat helped keep them up-to-date with the most recent knowledge in medical education. For example, “it helps me feel like I keep my finger on the pulse of MedEd.”

Besides positive comments, some respondents also indicated barriers to participation, with timing being the biggest issue. Besides, some mentioned that they were not used to how discussions take place on Twitter. As one mentioned, “I find it difficult to follow a conversation during the actual session as there are multiple threads happening at the same time. I find it somewhat easier to read a transcript when I can try to follow the thread of each conversation.” Other people find the format quite frustrating with “the short sentence format” and that one felt “Twitter is a mess with overlapping replies and reposts/retweets.” As some people may not be quite familiar with Twitter, one respondent further suggested that “it could perhaps be better…if some detailed step-by-step instructions as to how these sessions actually work via Twitter could be provided.” A summary of the gains from and barriers to participation in #MedEdChat is provided in Table 5.

**Table 5:** Gains from and barriers to participating in #MedEdChat

	Themes	Examples
Gains	Resource sharing	“The resource list that is noted at the beginning of the transcript has helped me so much.”
	New ideas and opinions	“I become aware of the interests of others and the topics that are at the front of educator’s minds.”
	Sense of community	“For me it’s all about community – we get to reach outside our silos (whether that’s institutional, clinical specialty, or geography)”
	Connection making	“Connections hearing from people I admire but not personally connected with”
Barriers	Timing issue	“It would be great if it were earlier in the evening sometimes! 9 pm is too late for me usually …”
	Technological difficulty	“I’m not very comfortable/familiar with Twitter … it could perhaps be better if the sessions were offered via a different, easier to follow platform, or if some detailed step-by-step instructions as to how these sessions actually work via Twitter could be provided.”

## Discussion

It is interesting to find that while people perceive the #MedEdChat discussions and transcripts helpful for resource sharing, learning new ideas and opinions, and building a sense of community for like-minded professionals, most people tend to be lurkers (who only read tweets or transcripts), like or retweet others’ tweets, rather than posting their own original tweets. This is consistent with other research that lurkers usually compose the largest population on social media such as Twitter [31], as well as the so called 90-9-1 rule in social media usage, that 90% of users are lurkers (i.e., read transcripts or observe discussions, but don’t contribute), 9% of the users contribute from time to time, and 1% of users participate a lot and account for most contributions [20]. It is also consistent with previous research suggesting that most virtual communities consist of a mix of lurkers, observers, and passive and active contributors [14, 23]. This does not necessarily mean that lurkers do not benefit from this learning community. Reading post-discussion transcripts could also increase knowledge uptake [4, 19]. However, since more senior physicians or educators have been described as inherent “slow adopters” of emerging technologies including Twitter, and we do not want to have their voices missed out in Twitter conversations, we should think about ways to encourage and facilitate their participation. As the barrier for participation could be unfamiliarity with the format of Twitter or being uncomfortable with a flood of short messages, providing novice Twitter users with guidance on how to participate can potentially help encourage new participants [4], and help them transition from knowledge consumers to knowledge producers. In addition, based on our observation that few residents or fellows were involved in the focal conversation, it would appear important for more medical educators to circulate such conversations among senior practitioners as well as other medical educators, and thus allow the main body of participants to hear a broad array of voices and perspectives.

In terms of what influenced participants’ satisfaction on a Twitter-supported professional learning community, our study suggested that sense of connectedness and cognitive presence significantly predicted Twitter users’ satisfaction. The more they felt connected to other participants, developed a sense of trusting and a spirit of community, the more satisfied participants are towards #MedEdChat. The significant effect of cognitive presence suggested that the topics and questions posted need to be intriguing enough to allow participants share different perspectives, construct explanations, and apply the knowledge into their own work.

Although moderator presence did not show a significant effect on satisfaction, participants rated it the highest among three presences. In #MedEdChat, the moderator played a significant role in creating an open, welcoming, and safe environment to ensure meaningful discourse and moving discussions towards the right direction. The moderator would welcome new participants and introduce people into the discussion. For every discussion, he would post three guiding questions (each one under discussion for approximately 20 minutes) throughout the session to facilitate the discussion. For example, in the discussion topic titled “Train faculty to teach in learner-centered environment”, the moderator posted three guiding questions gradually throughout the session to guide the discussion: 1. What do we mean by learner-centered teaching? 2. Are there new and emerging uses of educational technology that could be harnessed for learner-centered teaching? 3. How important is reflection in learner-centered education? These guiding questions could ensure the sequence and depth of discussion getting deeper with time going on. As previous research suggested, 70% of the variance in cognitive presence can be accounted for by social presence and teaching presence [26], thus it is important to emphasize moderator’s facilitative role in engaging participates in the meaningful and worthwhile discussions in this professional learning community.

It is surprising to find that participants rated social presence the lowest compared to cognitive presence and moderator presence, especially considering Twitter as a social platform. The finding is also contradictory to previous research suggesting a strong social presence on Twitter-facilitated discussion among students [3, 21, 27]. The lowest rated one among social presence was “I feel comfortable participating in the #MedEdChat discussions”. One possible explanation is that since almost half of the surveyed respondents rated themselves as lurkers, those were exactly the population who felt less comfortable with either the format of Twitter or with the way discussions take place on Twitter. For those lurkers who mostly read the transcripts, it is of course the case they felt less social presence since they did not directly involve in the social interactions on #MedEdChat. Nevertheless, for facilitators who moderate Twitter-facilitated online discussions, it is important to think of ways to facilitate social presence, even though Twitter has the inherent social functions. For example, facilitators could encourage people to introduce themselves when newly joining the discussion. They could also model open communication and free expression of emotions by posting tweets demonstrating how they like others’ comments, using humor, or bringing their personal experience into discussions.

## Limitations

Our study is not without limitations. First, we distributed the survey through the DR-ED listserv and through #MedEdChat discussions that took place in December 2020. Without knowing the population of participants on #MedEdChat, it is impossible for us to calculate the response rate. The number of respondents is nevertheless small, relative to the broad audience of Twitter. The results could thus be biased toward the particular population who were willing to complete the survey. Second, our sample size was comparatively small for path analysis. While our study does meet the criteria of what Kline [15] has suggested of a 10:1 ratio for sample size (*N* = 68) to parameters (N = 6), the ideal ratio would be 20:1; and of course, with a larger sample size, the analysis would yield greater explanatory power. Finally, this study asked about participants’ community of inquiry through attitudinal survey. Future research could conduct content analysis of Twitter posts to more accurately capture the three presences existing in Twitter-facilitated discussions [3].

## Conclusions

As the rising generation of future health professionals has grown up with social media, it is foreseeable that such media will be integrated into educational spaces and professional-development initiatives [17]. Our study found that most participants in #MedEdChat on Twitter were lurkers who chose to read the transcripts afterwards, or who liked or retweeted others’ tweets. Overall, participants were quite satisfied with #MedEdChat as a professional-learning community for medical educators, and expressed highly positive views of the moderators’ facilitating role. In addition, cognitive presence (i.e., the knowledge-construction process) and sense of connectedness were found to make significant positive contributions to the participants’ satisfaction with Twitter discussions associated with this hashtag. While social presence and moderator presence did not exhibit significant effects, we feel that creating an open and risk-free environment is indispensable for building a sense of trust among community members and moderator plays a significant role in achieving that. This study’s findings further suggest that we should encourage more active participation by providing guides to new joiners and create an open atmosphere for people who are not familiar and comfortable with the format of Twitter.

## Data Availability

The datasets generated and analyzed during the current study are available in the OSF repository, https://osf.io/wkad4/?view_only=c2e813df92b2417391e9773c7c050ff2.

## Abbreviations

CoP: Community of practice
CoI: Community of inquiry
WBL: Work-based learning
